# Early Postnatal Growth and Pubertal Timing: An Ultrasound-Based Study of Breast and Testicular Development

**DOI:** 10.1210/jendso/bvaf191

**Published:** 2025-11-25

**Authors:** Melissa Rajini Balthasar, Mathieu Roelants, Bente Brannsether-Ellingsen, Jennifer Lyn Baker, Dorthe Corfitzen Pedersen, Ingvild Særvold Bruserud, Ingvild Halsør Forthun, Nina Iszatt, Petur Benedikt Juliusson

**Affiliations:** Department of Pediatrics and Adolescents, Stavanger University Hospital, Stavanger 4011, Norway; Department of Clinical Science, University of Bergen, Bergen 5020, Norway; Department of Public Health and Primary Care, University of Leuven, Leuven 3000, Belgium; Department of Pediatrics and Adolescents, Stavanger University Hospital, Stavanger 4011, Norway; Center for Clinical Research and Prevention, Copenhagen University Hospital–Bispebjerg and Frederiksberg, Frederiksberg, Copenhagen 2000, Denmark; Center for Clinical Research and Prevention, Copenhagen University Hospital–Bispebjerg and Frederiksberg, Frederiksberg, Copenhagen 2000, Denmark; Children and Youth Clinic, Haukeland University Hospital, Bergen 5021, Norway; Department of Clinical Science, University of Bergen, Bergen 5020, Norway; Children and Youth Clinic, Haukeland University Hospital, Bergen 5021, Norway; Department of Food Safety, Norwegian Institute of Public Health, Oslo 0213, Norway; Department of Clinical Science, University of Bergen, Bergen 5020, Norway; Children and Youth Clinic, Haukeland University Hospital, Bergen 5021, Norway; Department of Health Registry Research and Development, Norwegian Institute of Public Health, Bergen 5808, Norway

**Keywords:** postnatal growth, rapid growth, ultrasound, puberty, thelarche, testicular growth

## Abstract

**Context:**

Rapid postnatal weight gain has been associated with earlier puberty in girls, while evidence in boys is less consistent. However, studies using objective pubertal markers remain limited.

**Objective:**

This work aimed to examine associations between postnatal growth during early infancy (0-0.5 years), late infancy (0.5-2 years), and early childhood (2-4 years) and pubertal onset.

**Methods:**

A total of 610 healthy, term-born children (260 boys) aged 6 to16 years, from the Bergen Growth Study 2 (2016) underwent ultrasound-based assessments of breast development and testicular volume. Longitudinal length/height and weight data were retrospectively collected from child health centres. Growth trajectories were modeled using piecewise linear mixed-effects models. Logistic regression was used to analyze sex-specific associations between postnatal growth and ultrasound-measured breast stage ≥ 2 and menarche in girls, ultrasound-measured testicular volume ≥ 2.7 mL, and testosterone level ≥ 0.5 nmol/L in boys, and pubarche in both sexes.

**Results:**

In girls, increased linear growth and weight gain during infancy and early childhood were associated with earlier breast development. Increased weight and body mass index gain in early childhood were linked to earlier menarche. In boys, increased length gain in early infancy was associated with earlier testicular growth, and increased length and weight gain during later periods were associated with higher testosterone levels (all *P* < .05). No statistically significant associations were found for pubarche in either sex.

**Conclusion:**

Increased linear growth during early infancy was associated with earlier pubertal onset in both sexes, with more consistent associations for weight gain from late infancy, particularly in girls.

The timing of puberty represents a complex biological and physiological event manifesting through an interplay between genetic background and environmental factors [1, 2]. Over several decades, the secular trend toward earlier pubertal onset [3-6] has been attributed to improved nutrition and socioeconomic and living conditions [7, 8]. Early puberty has been linked to psychosocial challenges and adolescent risk-taking behavior [9, 10]. In addition, it may act as an independent risk factor or mediator in the development of adverse health outcomes, such as obesity, type 2 diabetes, and cardiovascular disease [11, 12].

Growth during infancy and childhood, characterized by tempo of growth and hormonal regulation [13], has been linked to the timing of puberty [14, 15]. Small birth size with catch-up or increased growth during infancy has been associated with pubertal milestones, such as earlier attainment of Tanner stages for breast, pubic or genital development, greater testicular volume, and earlier menarche [16-19]. Further, rapid growth during early childhood has also been associated with earlier timing of puberty, with more consistent associations in girls than boys [20-23]. Many of these studies rely on self-reported Tanner staging, which may be subject to misclassification, and on later-occurring pubertal markers like menarche or age at peak height velocity (APHV), limiting the ability to capture the timing of pubertal onset. It remains unclear which time periods of early growth most strongly influence pubertal onset. Identifying these sensitive periods could help guide early monitoring practices and interventions.

During clinical investigation, a combination of visual inspection and breast palpation is considered the gold standard to evaluate the presence of glandular breast tissue [24, 25]. In boys, the Prader orchidometer is used to manually assess testicular volume [26]. The present study is based on a pubertal reference study, the Bergen Growth Study 2 (BGS2), in which ultrasound was used to estimate the breast developmental stages and testicular volume [27]. Ultrasound imaging enables clinicians to differentiate between adipose tissue and mammary gland, detect nonpalpable glandular breast tissue, and distinguish between true testicular volume and surrounding scrotal tissue in boys [28, 29].

The aim of the present study was to investigate whether increased linear growth and weight gain during distinct early growth periods between birth and age 4 years are associated with pubertal onset in healthy, term-born children, using breast and testicular ultrasound imaging. Further, serum testosterone levels in boys, menarcheal status in girls, and pubarche in both sexes were included as additional pubertal markers.

We hypothesized that increased linear growth and weight gain during early infancy and early childhood were associated with earlier pubertal onset, with a more pronounced association in girls than in boys.

## Materials and Methods

### Study Population

The BGS2, a cross-sectional study conducted from January to June 2016 in children aged 6 to 16 years, aimed to evaluate pubertal development and body composition, characterized by somatic and endocrine changes [30-33]. In the present study, early-life growth data on length/height and weight were retrospectively collected from routine visits at child health centers for participants in the BGS2 cohort, of whom 847 out of 1171 (72.3%) provided informed consent. Information on child and maternal characteristics were obtained from parental questionnaires and used to identify and exclude children with conditions that could influence early growth. In the analytical sample, we included term-born singletons (37-42 weeks of gestation), with a birth weight of 2500 g or greater, and born to a mother without preeclampsia or gestational diabetes (Supplementary Fig. S1 [34]). In addition, 37 children were excluded due to chronic illness that could affect growth, and 18 boys were excluded due to scrotal pathology. This resulted in a cohort of 350 girls and 260 boys with data on early growth and pubertal assessment between ages 6 and 16 years. Age boundaries for defining pubertal onset were based on previously published reference curves reflecting normative timing of ultrasound-assessed breast development and testicular volume, as well as pubic hair development [30, 31]. Hence, only girls between ages 8 and 13 years (n = 206), and boys between ages 9 and 14.5 years (n = 146) were selected to avoid inclusion of ages during which all children were prepubertal or all were in puberty. Analyses of menarche (n = 144) were based on girls aged 11 to 16 years.

### Early-Life Growth Data

Growth measurements are routinely performed by health-care personnel during scheduled health check-ups at Norwegian child health centres and school health services, including 14 routine consultations between birth and age 5 years, and additional visits at age 6 and 8 years [35]. On average, 15 measurements were available per child from birth to 8 years of age. Anthropometric measurements from routine visits were used to calculate sex- and age-adjusted *z*-scores (SD scores) for length/height, weight, and body mass index (BMI), based on Norwegian reference data [36]. According to national guidelines, length is measured recumbently until age 2 and standing thereafter, recorded to the nearest 0.1 cm. Weight is recorded to the nearest 10 g for children up to age 2, and to the nearest 100 g for children older than 2 years [35]. Changes in *z*-scores for length/height, weight, and BMI were estimated across 3 age periods: early infancy (age 0-0.5 years), late infancy (age 0.5-2 years), and early childhood (age 2-4 years). These age intervals were selected based on the standard schedule of routine measurements and their alignment with distinct hormonal environments [13].

### Pubertal Markers by Clinical Examination and Ultrasound Assessment

In BGS2, examinations were conducted during school hours at the child's school. In girls, a trained examiner performed the clinical examination according to Tanner stages [24] and ultrasound-measured breast stage (USB), following a protocol described in a previous publication [28]. Ultrasound evaluation of the breast was performed with a SonoSite Edge (FUJIFILM SonoSite) device with a 15- to 16-MHz (5-cm) linear transducer. Girls were classified as prepubertal or pubertal, with USB ≥ 2 indicating pubertal onset (thelarche), characterized by a hyperechoic retroareolar glandular breast tissue with a round or star-shaped hypoechoic center. Two girls had missing ultrasound data, and this was replaced by Tanner B in the analyses. Ultrasound and Tanner staging of breast development have demonstrated strong concordance (Cohen κ = 0.87; κ = 0.94 when dichotomized at thelarche), with ultrasound detecting thelarche slightly earlier on average [30]. Menarcheal status (yes/no) was also assessed during the clinical examination.

In boys, a trained radiographer performed the ultrasound examination of the right testicle according to a standardized protocol [37]. Briefly, the dimensions of the right testicle were recorded, and the testicular volume was calculated using the Lambert equation (length × width × depth × 0.71). The intraobserver variability was 9.2% and the technical error of measurement was 6.5% [37]. An empirical equation to predict the equivalent Prader orchidometer volume from ultrasound volume was previously derived using the nonlinear formula Vol_OM_ = 1.96 × Vol_US_^0.71^. Boys were classified as prepubertal or pubertal, with ultrasound-measured testicular volume (USTV) ≥ 2.7 mL corresponding to a Prader orchidometer volume of 4 mL or greater, defining pubertal onset [37]. A strong correlation between circulating testosterone levels and testicular volume, as measured by ultrasound, has previously been demonstrated in pubertal boys [32]. A nonfasting blood sample was collected between 8 Am and 2 Pm, and total testosterone was assayed by liquid chromatography–tandem mass spectrometry at the Hormone Laboratory at Haukeland University Hospital in Bergen, Norway, using a previously published method [38]. A testosterone level ≥ 0.5 nmol/L was used as an alternative marker of pubertal onset, based on a previously published receiver operating characteristic analysis in boys from the BGS2 cohort [39]. Serum estradiol, follicle-stimulating hormone (FSH), and luteinizing hormone (LH) were also analyzed as described previously [33]. However, no comparable cutoff is established for estradiol due to biological variability among girls, and LH and FSH levels were limited by diurnal variation [40]. Assessment of Tanner pubic hair stage (Tanner PH) both in girls and boys was visually assessed in the supine position based on descriptions by Marshall and Tanner, whereby Tanner PH ≥ 2 defined pubarche [24, 41]. Tanner PH stage was included in the protocol for boys from the start and systematically assessed in girls from February 22 onward.

### Anthropometric Measurements During Pubertal Development

Height was measured in the standing position with a Harpenden Portable Stadiometer (Holtain Ltd) and recorded to the nearest 0.1 cm. Weight was measured in light clothing with an electronic scale (Tanita MC-780MA, Tanita Corp of America Inc) with a precision of 0.1 kg [30]. BMI was calculated as weight in kilograms divided by height in meters squared. Weight status was categorized according to the International Obesity Task Force (IOTF) criteria [42]. Height, weight, BMI, waist circumference (WC), and subscapular skinfold (SSF) were converted to *z*-scores using national growth charts [36, 43, 44].

### Covariates

Information on maternal characteristics such as prepregnancy weight, weight gain during pregnancy, height, smoking during pregnancy, parity, and age at menarche was obtained through a written questionnaire. The highest parental educational level was used as an indicator of socioeconomic status and classified as primary education (9 years of school), secondary education (12 years of school), or higher education (15 years or more). Confounders that have been previously associated with infant growth and pubertal development [19] were adjusted for, and these confounders were also identified using a directed acyclic graph prior to the analysis (Supplementary Fig. S2 [34]). Current BMI was considered a potential collider, since both postnatal growth and pubertal timing are determinants of BMI at the time of assessment, and adjusting for it could therefore introduce bias. Maternal prepregnancy BMI was calculated by subtracting total gestational weight gain from weight at the end of pregnancy and dividing the result by height in meters squared. All covariates were dichotomized and included maternal prepregnancy BMI (≥25/<25), maternal age at menarche (≤12 years/>12 years), parity (first born/later born), socioeconomic status (college or university/secondary or lower), and prenatal smoking (yes/no). Description of the study population by pubertal status among girls and boys included in the analysis of pubertal onset is presented in Table 1.

**Table 1.** Characteristics of the study population by pubertal status among girls (age 8-13 years) and boys (age 9-14.5 years) included in the analyses of pubertal onset (n = 352, Bergen Growth Study 2, 2016)

	Girls		Boys	
Characteristics	Prepubertal USB < 2 (n = 99)	Pubertal USB ≥ 2 (n = 107)	Prepubertal USTV < 2.7 mL (n = 86)	Pubertal USTV ≥ 2.7 mL (n = 60)
Birth weight, kg (SD)	3.6 (0.4)	3.6 (0.4)	3.8 (0.4)	3.7 (0.6)
Birth length, cm (SD)	50.4 (1.8)	50.2 (1.9)	51.5 (1.7)	50.9 (2.3)
**Maternal prepregnancy BMI**				
Underweight (BMI < 18.5)	4 (4.9%)	4 (4.7%)	5 (8.3%)	0
Normal weight (BMI ≥ 18.5-<25)	63 (77.8%)	66 (76.7%)	44 (73.3%)	33 (82.5%)
Overweight (BMI ≥ 25.0-<30)	12 (14.8%)	14 (16.3%)	10 (16.7%)	7 (17.5%)
Obesity (BMI ≥ 30.0)	2 (2.5%)	2 (2.3%)	1 (1.7%)	0
Missing	18 (18.2%)	21(19.6%)	26 (30.2%)	20 (33.3%)
**Smoking during pregnancy**				
Yes	5 (5.1%)	9 (8.5%)	4 (4.8%)	3 (5.0%)
No	93 (94.9%)	97 (91.5%)	80 (95.2%)	57 (95.0%)
Missing	1 (1.0%)	1 (0.9%)	2 (2.3%)	0
**Parity**				
First born	43 (43.4%)	53 (51.0%)	36 (41.9%)	23 (38.3%)
Second or later born	55 (55.6%)	51 (49.0%)	50 (58.1%)	37 (61.7%)
Missing	1 (1%)	3 (2.8%)	0	0
**Maternal age at menarche, y**				
≤12	21 (25.0%)	27 (29.3%)	20 (28.2%)	18 (34.0%)
>12-13	21 (25.0%)	35 (38%)	20 (28.2%)	15 (28.3%)
>13	42 (50.0%)	30 (32.6%)	31 (43.7%)	20 (37.7%)
Missing	15 (15.2%)	15 (14.0%)	15 (17.4%)	7 (11.7%)
**Parental educational level, y**				
No secondary education, 9	1 (1.0%)	0	1 (1.2%)	3 (5.0%)
Secondary education, 12	8 (8.1%)	19 (17.9%)	11 (12.9%)	12 (20.0%)
College/university, ≥15	90 (90.9%)	87 (82.1%)	73 (85.9%)	45 (75.0%)
Missing	0	1 (0.9%)	1 (1.2%)	0
**Parental origin**				
Norwegian	85 (88.5%)	86 (83.5%)	66 (80.5%)	46 (80.7%)
European	8 (8.3%)	7 (6.8%)	7 (8.5%)	4 (7.0%)
Non-European	3 (3.1%)	10 (9.7%)	9 (11.0%)	7 (12.3%)
Missing	3 (3.0%)	4 (3.7%)	4 (4.7%)	3 (5.0%)

Continuous variables are presented as means ± SD, and categorical variables are presented as numbers and percentages. Within-sex comparisons showed no statistically significant differences, with all *P* values greater than .05. Independent-samples *t* tests and Pearson chi-square tests were used, as appropriate.

Abbreviations: BMI, body mass index; USB, ultrasound-measured breast stage; USTV, ultrasound-measured testicular volume.

### Statistical Analysis

Descriptive statistics are presented as means (±SD) and medians (interquartile range, IQR) for continuous variables, and as counts and percentages for categorical variables. Comparisons of characteristics, hormone levels, and anthropometric measures within sexes were analyzed using independent-sample *t* tests or Mann-Whitney *U* tests for continuous variables, and Pearson chi-square tests for categorical variables, as appropriate (see Table 1 and Table 2). Growth trajectories were estimated using a piecewise linear mixed-effects model, including children with measurements at birth (weight and length) and at least 1 additional measurement up to age 8. The knots were placed at target ages 0, 0.5, 2, 4, 6, and 8 years. The model included random slopes for time to account for individual variation in growth trajectories, together with fixed effects for the population mean. This approach allowed for the prediction of growth measures at the target ages, accommodating variation in the timing of routine assessments and handling missing data at some visits. Associations between early growth and pubertal markers were analyzed using binary logistic regression. Separate models were run for each age interval and growth measure to estimate the odds ratio (OR) of having reached pubertal onset defined as USB ≥ 2 in girls, USTV ≥ 2.7 mL or serum testosterone ≥ 0.5 nmol/L in boys, as well as for Tanner PH ≥ 2, and menarche (yes/no). In the crude analysis, model 1, we adjusted for baseline *z*-score at the start of each age interval and age at pubertal assessment. For example, when analyzing weight gain between age 0.5 and 2 years, we adjusted for a weight *z*-score at 0.5 years to account for baseline growth status, reflecting birth outcomes and earlier postnatal growth. In model 2, we included additional adjustments for potential confounders. Birth weight may influence both postnatal growth [45] and pubertal timing, either directly [17] or indirectly through its effect on early growth trajectories [21]. To avoid overadjustment, birth weight was not included in the analyses of growth intervals at later ages, as it precedes the exposure and may confound associations through their links with other covariates. However, to assess its possible effect it was included in sensitivity analyses. Missing covariates and outcomes, including Tanner PH and testosterone (see Tables 1 and 2), were imputed with plausible values based on the distributions and relationships among the observed nonmissing data using multiple imputation by chained equations [46], generating 20 imputation sets. Model 1 was based on observed data. However, model 2 estimates were derived by pooling results from the multiply imputed datasets, thereby accounting for uncertainty resulting from the imputation of missing data. Statistical analyses were conducted using R software (version 4.4.1), using the “lme4” package for mixed-effects modeling and the “mice” package for multiple imputation by chained equations. In all analyses, a *P* value less than .05 was considered statistically significant.

**Table 2.** Hormone levels and anthropometry in girls (age 8-13 years) and boys (age 9-14.5 years) before or after pubertal onset at the time of examination (n = 352, Bergen Growth Study 2, 2016)

	Girls		Boys	
	Prepubertal USB < 2 (n = 99)	Pubertal USB ≥ 2 (n = 107)	Prepubertal USTV < 2.7 mL (n = 86)	Pubertal USTV ≥ 2.7 mL (n = 60)
Age (IQR), y	9.4 (1.46)	11.3 (1.71)*^a^*	10.2 (1.34)	12.6 (1.46)*^a^*
Testosterone ≥ 0.5 nmol/L	1 (1.1%)	42 (41.6%)*^a^*	3 (3.8%)	49 (92.5%)*^a^*
Testosterone, nmol/L (IQR)	0.1 (0.1)	0.4 (0.4)*^a^*	0.1 (0.1)	4.1 (8.4)*^a^*
Missing	4 (4.0%)	6 (5.6%)	6 (7.0%)	7 (11.7%)
Estradiol, pmol/L (IQR) Missing	6.2 (8.3) 8 (8.1%)	61.0 (110.0)*^a^* 15 (14.0%)	1.1 (1.2) 8 (9.3%)	11.5 (34.5)*^a^* 10 (16.7%)
FSH, IU/L (IQR) Missing	1.7 (1.3) 4 (4.0%)	4.7 (4.1)*^a^* 6 (5.6%)	1.3 (1.1) 6 (7.0%)	2.8 (2.2)*^a^* 7 (11.7%)
LH, IU/L (IQR)	0.1 (0.1) 4 (4.0%)	1.4 (2.5)*^a^* 6 (5.6%)	0.2 (0.3) 7 (8.1%)	1.6 (1.0)^a^ 8 (13.3%)
Tanner PH ≥ 2	3 (5.0%)	41 (67.2%)*^a^*	11 (13.1%)	43 (72.8%)*^a^*
Missing	39 (39.4%)	46 (43%)	2 (2.3%)	1 (1.7%)
Height *z* score (SD)	−0.15 (1.06)	0.49 (1.06)*^a^*	−0.09 (0.92)	0.15 (1.04)
Weight *z* score (SD)	−0.38 (0.85)	0.37 (0.96)*^a^*	−0.18 (0.93)	0.00 (0.91)
BMI *z* score (SD)	−0.40 (0.85)	0.19 (1.00)*^a^*	−0.15 (0.93)	−0.12 (0.87)
WC *z* score (SD)	−0.25 (0.82)	0.25 (0.91)*^a^*	−0.07 (0.83)	0.04 (0.86)
SSF *z* score (SD)	−0.36 (0.99)	0.13 (1.06)*^a^*	0.03 (0.96)	−0.19 (1.09)
Missing	0	1 (0.9%)	0	0
IOTF ≥ 25	5 (5.1%)	20 (18.7%)*^a^*	7 (8.1%)	2 (3.3%)

Means are presented with SD, medians with IQR, and numbers with percentages.

Abbreviations: BMI, body mass index; FSH, follicle-stimulating hormone; IOTF, International Obesity Task Force; IQR, interquartile range; LH, luteinizing hormone; PH, pubic hair; SSF, subscapular skinfold; USB, ultrasound-measured breast stage; USTV, ultrasound-measured testicular volume; WC, waist circumference.

^a^Within-sex comparisons based on pubertal status with a *P* value less than .05. Independent-samples *t* tests, Mann-Whitney *U* tests, or Pearson chi-square tests were used as appropriate.

### Ethics Approval

Written informed consent was obtained from a parent or legal guardian of each participant in the study, as well as assent from the participants themselves. A cinema voucher was given as an incentive. The study was approved by the Regional Committee for Medical and Health Research Ethics West (REC-WEST 2015/128).

## Results

### Characteristics

A total of 206 girls and 146 boys were included in the analysis of pubertal onset, and Table 1 presents their characteristics stratified by pubertal status. Most children had parents with a college or university education, Norwegian ethnicity, mothers with a normal prepregnancy BMI, and who did not smoke during pregnancy. Table 2 presents within-sex comparisons of hormone levels and anthropometry based on pubertal status. The median age for girls with USB ≥ 2 was 11.3 years, the median age for boys with USTV ≥ 2.7 mL was 12.6 years. The median serum testosterone level in prepubertal boys was 0.10 nmol/L (IQR = 0.10) and 4.10 nmol/L in pubertal boys, with a much wider IQR of 8.4, reflecting substantial variability in hormone levels during the progression of puberty. Among the prepubertal girls, 3 of 60 (5%) had Tanner PH ≥ 2. Among the prepubertal boys, 11 of 84 (13.1%) were similarly classified as Tanner PH ≥ 2. Mean *z*-score of BMI, WC, and SSF were significantly higher in pubertal compared to prepubertal girls, but this was not observed between the pubertal and prepubertal boys.

### Association Between Early Growth and Pubertal Markers in Girls

Increased length, height, and weight gain in all 3 age periods (early infancy, late infancy, and early childhood) showed a positive association with pubertal onset, USB ≥ 2 between ages 8 and 13 years, most consistent across the 3 measures for early childhood, suggesting earlier pubertal onset compared to girls with slower growth (Table 3). An increase in length or height *z*-score of 1 SD from 0 to 0.5 years and 2 to 4 years was significantly associated with higher odds of having reached pubertal onset (USB ≥ 2) with adjusted odds ratios (AORs) of 1.94 (95% CI, 1.07-3.59) and 5.16 (95% CI, 1.49-19.23), respectively. An increase in weight *z*-score of 1 SD from 0.5 to 2 years and 2 to 4 years was also significantly associated with higher odds of having reached pubertal onset with AORs of 2.05 (95% CI, 1.19-3.76) and 6.36 (95% CI, 2.09-21.53), respectively. Similarly, an increase in BMI *z*-score of 1 SD from 2 to 4 years was significantly associated with pubertal onset with an AOR of 2.69 (95% CI, 1.24-6.16). The associations remained significant after adjustments for potential confounders (model 2).

**Table 3.** Logistic regression of pubertal onset (ultrasound-measured breast stage ≥ 2) in girls aged 8 to 13 years according to postnatal linear growth and weight gain

	Model 1*^a^* (n = 206 girls)			Model 2*^b^* (n = 206 girls)		
	AOR	95% CI	*P*	AOR	95% CI	*P*
Δ **Length/height *z*-score**						
Early infancy (0-0.5 y)	1.94	1.07-3.59	.**030**	1.90	1.01-3.58	.**047**
Late infancy (0.5-2 y)	1.92	0.88-4.32	.107	1.85	0.82-4.15	.133
Early childhood (2-4 y)	5.16	1.49-19.23	.**011**	6.67	1.71-25.89	.**006**
Δ **Weight *z*-score**						
Early infancy (0-0.5 y)	1.10	0.73-1.65	.642	1.07	0.70-1.63	.766
Late infancy (0.5-2 y)	2.05	1.19-3.76	.**014**	2.10	1.16-3.82	.**015**
Early childhood (2-4 y)	6.36	2.09-21.53	.**002**	6.94	2.06-23.37	.**002**
Δ **BMI *z*-score**						
Early infancy (0-0.5 y)	0.92	0.65-1.31	.662	0.91	0.63-1.31	.596
Late infancy (0.5-2 y)	1.47	0.92-2.43	.116	1.53	0.92-2.56	.102
Early childhood (2-4 y)	2.69	1.24-6.16	.**015**	2.75	1.20-6.33	.**017**

ORs reflect the change in odds of the outcome when the respective *z*-scores increase by 1 SD during the specified age interval. Estimates with *P* values less than .05 are shown in bold. USB ≥ 2 was observed in n = 107/206 girls.

Abbreviations: AOR, adjusted odds ratio; BMI, body mass index; OR, odds ratio; USB, ultrasound-measured breast stage.

^a^In model 1 we adjusted for baseline *z*-score and age at pubertal assessment.

^b^In model 2 we additionally adjusted for covariates and used multiple imputation by chained equations to impute missing data. Covariates: prepregnancy BMI (≥ 25), socioeconomic status (highest parental education), parity (multiparous), maternal menarcheal age (≤ 12 years), and prenatal smoking (yes).

Among girls aged 11 to 16 years, greater increases in weight and BMI *z*-scores from ages 2 to 4 years were significantly associated with higher odds of having reached menarche, with AORs of 5.14 (95% CI: 1.34-20.88) and 3.71 (95% CI: 1.30-11.15), respectively, both with and without adjustment for confounders (Table 4).

**Table 4.** Logistic regression of menarcheal status in girls aged 11 to 16 years according to postnatal linear growth and weight gain

	Model 1*^a^* (n = 144 girls)			Model 2*^b^* (n = 144 girls)		
	AOR	95% CI	*P*	AOR	95% CI	*P*
Δ **Length/height *z*-score**						
Early infancy (0-0.5 y)	1.40	0.65-3.08	.399	1.64	0.64-4.17	.295
Late infancy (0.5-2 y)	1.58	0.63-4.08	.332	1.47	0.52-4.13	.462
Early childhood (2-4 y)	2.68	0.63-11.97	.186	2.72	0.44-16.67	.277
Δ **Weight *z*-score**						
Early infancy (0-0.5 y)	0.96	0.54-1.68	.880	1.01	0.53-1.95	.973
Late infancy (0.5-2 y)	1.00	0.49-2.03	.990	0.93	0.42, 2.09	.864
Early childhood (2-4 y)	5.14	1.34-20.88	.**018**	7.67	1.53- 38.54	.**014**
Δ **BMI *z*-score**						
Early infancy (0-0.5 y)	0.88	0.55-1.39	.580	0.92	0.55-1.54	.752
Late infancy (0.5-2 y)	0.60	0.29-1.18	.154	0.61	0.28-1.37	.233
Early childhood (2-4 y)	3.71	1.30-11.15	.**015**	4.22	1.24-14.36	.**022**

Menarche was reached in n = 79/144 girls. ORs reflect the change in odds of the outcome when the respective *z*-scores increased by 1 SD during the specified age interval. Estimates with *P* values less than .05 are shown in bold.

Abbreviations: AOR, adjusted odds ratio; BMI, body mass index; OR, odds ratio.

^a^In model 1 we adjusted for baseline *z*-score and age at pubertal assessment.

^b^In model 2 we additionally adjusted for covariates and used multiple imputation by chained equations to impute missing data. Covariates: prepregnancy BMI (≥25), socioeconomic status (highest parental education), parity (multiparous), maternal menarcheal age (≤12 years), and prenatal smoking (yes).

Increased length, height, and weight gain in all 3 age periods showed a positive association with reaching Tanner PH ≥ 2, but the association was statistically significant only for the change in length *z*-score from age 0 to 0.5 years. However, this association was no longer statistically significant after adjusting for confounders and imputing missing data on Tanner PH stage (Supplementary Table S1 [34]).

### Association Between Early Growth and Pubertal Markers in Boys

Increased length, height, and weight gain during all 3 age periods showed a positive association with pubertal onset, USTV ≥ 2.7 mL between ages 9 and 14.5 years at the time of assessment (Table 5). Notably, a statistically significant association was only found for increased change in length *z*-score from ages 0 to 0.5 years (AOR: 2.82; 95% CI, 1.25-6.94), which persisted after adjusting for confounders. An increase in length *z*-score of 1 SD from ages 0.5 to 2 years and weight *z*- score from ages 2 to 4 years were significantly associated with higher odds of reaching the testosterone level, with AORs of 2.66 (95% CI, 1.06-7.34) and 7.07 (95% CI, 1.18-53.9), respectively (Table 6). After adjusting for confounders and imputing missing data on testosterone, the associations were no longer evident. There was little evidence of associations between length, height, and weight gain in any of the 3 age periods and reaching Tanner PH ≥ 2 (Supplementary Table S2 [34]).

**Table 5.** Logistic regression of pubertal onset (ultrasound-measured testicular volume ≥ 2.7 mL) in boys aged 9 to 14.5 years according to postnatal linear growth and weight gain

	Model 1*^a^* (n = 146 boys)			Model 2*^b^* (n = 146 boys)		
	AOR	95% CI	*P*	AOR	95% CI	*P*
Δ **Length/height *z*-score**						
Early infancy (0-0.5 y)	2.82	1.25-6.94	.**017**	2.83	1.16-6.95	.**024**
Late infancy (0.5-2 y)	1.79	0.73-4.48	.201	1.37	0.51-3.72	.532
Early childhood (2-4 y)	3.13	0.34-32.16	.318	3.94	0.30-51.27	.292
Δ **Weight *z*-score**						
Early infancy (0-0.5 y)	1.46	0.77-2.84	.249	1.32	0.67-2.60	.415
Late infancy (0.5-2 y)	1.25	0.53-3.00	.604	1.26	0.50-3.18	.629
Early childhood (2-4 y)	5.67	1.04-37.27	.055	6.63	0.94-46.93	.058
Δ **BMI *z*-score**						
Early infancy (0-0.5 y)	1.07	0.59-1.90	.826	0.94	0.52-1.71	.841
Late infancy (0.5-2 y)	0.92	0.44-1.91	.826	0.99	0.45-2.22	.989
Early childhood (2-4 y)	1.66	0.63-4.72	.321	1.85	0.62-5.57	.269

ORs reflect the change in odds of the outcome when the respective *z*-scores increased by 1 SD during the specified age interval. Estimates with *P* values less than .05 are shown in bold. USTV ≥ 2.7 mL was observed in n = 60/146 boys.

Abbreviations: AOR, adjusted odds ratio; BMI, body mass index; OR, odds ratio; USTV, ultrasound-measured testicular volume.

^a^In model 1 we adjusted for baseline *z*-score and age at pubertal assessment.

^b^In model 2 we additionally adjusted for covariates and used multiple imputation by chained equations to impute missing data. Covariates: pr-pregnancy BMI (≥25), socioeconomic status (highest parental education), parity (multiparous), maternal menarcheal age (≤12 years), and prenatal smoking (yes).

**Table 6.** Logistic regression of pubertal onset (testosterone ≥ 0.5 nmol/L) in boys aged 9 to 14.5 years according to postnatal linear growth and weight gain

	Model 1*^a^* (n = 133 boys)			Model 2*^b^* (n = 146 boys)		
	AOR	95% CI	*P*	AOR	95% CI	*P*
Δ **Length/height *z*-score**						
Early infancy (0-0.5 y)	1.87	0.81-4.54	.150	1.70	0.72-4.01	.219
Late infancy (0.5-2 y)	2.66	1.06-7.34	.**043**	1.76	0.58-5.36	.313
Early childhood (2-4 y)	2.25	0.20-26.74	.509	.62	0.03-11.71	.743
Δ **Weight *z*-score**						
Early infancy (0-0.5 y)	1.24	0.63-2.51	.532	1.01	0.52-1.96	.970
Late infancy (0.5-2 y)	1.27	0.51-3.21	.604	1.23	0.51-2.99	.640
Early childhood (2-4 y)	7.07	1.18-53.9	.**042**	4.73	0.79-28.33	.089
Δ **BMI *z*-score**						
Early infancy (0-0.5 y)	1.16	0.63-2.14	.624	0.93	0.49-1.78	.833
Late infancy (0.5-2 y)	0.72	0.32-1.57	.416	0.73	0.35-1.58	.425
Early childhood (2-4 y)	2.65	0.91-8.77	.089	2.98	0.98-9.09	.055

Odds ratios reflect the change in odds of the outcome when the respective z-scores increase by 1 SD during the specified age interval. Estimates with *P* values less than .05 are shown in bold. Testosterone ≥ 0.5 nmol/L was observed in n = 52/133 boys.

Abbreviations: AOR, adjusted odds ratio; BMI, body mass index; OR, odds ratio.

^a^In model 1 we adjusted for baseline *z*-score and age at pubertal assessment.

^b^In model 2 we additionally adjusted for covariates and use multiple imputation by chained equations to impute missing data. Covariates: prepregnancy BMI (≥25), socioeconomic status (highest parental education), parity (multiparous), maternal menarcheal age (≤12 years), and prenatal smoking (yes).

### Sensitivity Analyses

Sensitivity analyses including adjustment for birth weight in the age intervals 0.5 to 2 years and 2 to 4 years did not materially affect the associations in both sexes (data not shown).

## Discussion

We examined whether increased linear growth and weight gain during distinct postnatal periods between early infancy, from ages 0 to 0.5 years, late infancy from ages 0.5 years to 2 years, and early childhood from 2 to 4 years were associated with pubertal timing in healthy, term-born children. We found statistically significant associations between greater linear growth during early infancy and pubertal onset in both sexes. Additionally, greater weight gain during late infancy and early childhood was also associated with earlier thelarche and menarche in girls. The novel use of ultrasound to assess pubertal onset represents a robust methodology that enhances objectivity compared to standard clinical evaluation.

In this healthy, term-born cohort, increased gain in length during early infancy was significantly associated with earlier pubertal onset in both sexes. Similar findings were also reported by Liimatta et al [21], who examined pubertal development in a Finnish cohort of children aged 9 to 11 years. However, they found statistically significant associations only among girls, which may be attributable to the early age range, possibly before many boys had entered puberty . In contrast, Wohlfahrt-Veje et al [18], who examined rapid linear growth from birth to 18 and 36 months in a Danish cohort, did not observe significant associations with early markers (B2 and TV >3 mL), but instead reported significant associations with later pubertal milestones such as B4, menarche, and G4. The exact mechanisms linking rapid early linear growth to earlier puberty are not completely understood. One possibility is genetic influence, which we partially accounted for by adjusting for maternal age at menarche. A recent review has reported that accelerated growth tempo in infancy may reflect an overall faster developmental trajectory, driven by early activation of the growth hormone/insulin-like growth factor-1 axis, potentially leading to earlier pubertal onset [47].

In girls, we found a statistically significant independent association between increased weight gain during late infancy and earlier pubertal onset, suggesting that late infancy may represent a sensitive window for pubertal programming. This aligns with findings from a Danish study [17] that reported that an increase in weight *z*-score between 5 and 12 months was more strongly associated with pubertal timing than growth between 0 and 5 months. In our study, increased weight gain during early childhood, from 2 to 4 years, was also associated with earlier pubertal onset in both sexes, as indicated by ultrasound measured breast stage in girls and testicular volume, as well as testosterone level in boys. Although the association between increased weight gain in this period and USTV ≥ 2.7 mL in boys did not reach statistical significance, possibly due to smaller sample size, we think it is biologically relevant due to the established relationship between testicular volume and circulating testosterone levels [32, 39]. The larger AOR and wider CI observed for the 2- to 4-year interval probably reflect smaller variations in *z*-score changes during this period (Supplementary Figs. S3A and S3B [34]). A 1-unit change in *z*-score is relatively uncommon, but when it occurs, it is typically associated with higher odds of pubertal onset, contributing to the observed effect size and uncertainty.

All models included baseline growth status at the start of each age interval, which reflects both birth size and postnatal growth during earlier periods. Sensitivity analyses adjusting for birth weight *z*-scores in the 6-month to 2-year and 2- to 4-year intervals yielded similar results, suggesting that the observed associations are not only attributable to differences in birth size. Furthermore, baseline growth status at the start of each interval was already included to account for initial size.

Our findings indicate greater consistency in girls than boys and align with previous studies that have examined specific periods of early growth in relation to the timing of puberty [20-22]. However, methodological differences limit direct comparisons across studies. In contrast to these studies, we did not find statistically significant associations between weight gain during the first 6 months of life and pubertal timing. Aris et al [20] reported that faster weight and BMI gain between ages 0 and 0.5 years was associated with earlier APHV in boys, while Liimatta et al [21] found that increased weight gain during the same period was associated with higher odds of adrenarche, pubarche, and thelarche. In addition to methodological differences, the discrepancies may also be explained by differences in cohort composition, birth size distribution, and sample size. We observed a significant association with the attainment of menarche only for weight and BMI gain between ages 2 and 4 years, but not during infancy, as previously reported [16, 21, 22, 48]. In the present study, we examined the influence of early postnatal growth in a low-risk healthy cohort by excluding preterm and low birthweight children, as well as children born to mothers with conditions known to affect fetal growth, such as gestational diabetes and preeclampsia. In addition, children with chronic illnesses that could affect growth were excluded. Early infancy reflects a transition from the prenatal environment, and the limited association between early infancy weight gain and pubertal onset may reflect the more homogeneous and moderate growth patterns expected in this healthy, low-risk cohort.

Rapid early weight gain is a well-established risk factor for childhood obesity [45], which in turn has been consistently linked to an earlier onset of puberty [49-51], suggesting a potential pathway through which early growth may influence pubertal timing. The putative mechanisms underlying this association have been previously described [15] and suggest that increased adiposity may lead to earlier sex steroid exposure through pathways involving insulin resistance, decreased sex hormone–binding globulin, and enhanced aromatase activity.

Pubarche and central puberty are two distinct developmental processes with different regulatory mechanisms. Increased linear growth during early infancy was associated with earlier pubarche in girls but lost statistical significance after covariate adjustment and inclusion of imputed data, suggesting that the association may be confounded by other factors or limited robustness due to missing data. There were no statistically significant associations between early growth and pubarche in boys, results also found by others [20, 21].

A systematic review concluded that higher BMI trajectory already during early childhood was linked to advanced puberty in girls [52], but in boys the results were inconclusive. The endocrine regulation of pubertal onset in girls and boys involves gonadotropins, sex steroids, and metabolic hormones such as leptin and ghrelin, and differs by sex [53]. During puberty, girls tend to accumulate a higher percentage of body fat compared to boys, who typically gain more lean body mass [54]. In our study there were statistically significant differences between mean *z*-scores of BMI, WC, and SSF between prepubertal and pubertal girls but not in boys, illustrating the presence of sexual dimorphism in pubertal development. Future longitudinal studies are needed to explore sex-specific differences in sensitivity to early growth and their relationship with pubertal onset. Ideally, such studies should incorporate repeated objective clinical assessments, alongside measurements of reproductive hormones and metabolic regulators such as insulin-like growth factor-1, insulin, leptin and adiponectin.

A major strength of the present study was the use of a robust and objective method for assessing pubertal onset. Ultrasound imaging offers greater precision in detecting glandular breast tissue and testicular volume, while minimizing misclassification by excluding adipose tissue and surrounding scrotal tissue. An additional strength is the inclusion of multiple pubertal markers enhancing the validity of the findings.

Postnatal growth was assessed longitudinally prior to the assessment of pubertal outcomes, which were measured cross-sectionally at one time point. While this temporality supports a potential causal relationship, causality cannot be definitively established due to the observational design. However, findings from sibling-matched studies suggest that greater postnatal weight gain is associated with earlier attainment of Tanner stages and menarche, supporting a causal link [48, 49]. There are well-described ethnic differences in the onset of puberty [51], and our findings, largely limited to children with Norwegian ethnicity, limit generalizability to other populations. Covariates such as maternal weight gain and maternal age at menarche were obtained from self-reported questionnaires, which may be subject to differential recall or misreporting, potentially introducing bias. We were able to adjust for several important confounders, however, the possibility of residual confounding cannot be excluded. Unmeasured factors such as maternal or child diet, physical activity, psychosocial stressors, or other prenatal influences may still affect the observed associations. Future research exploring behavioral factors such as diet, physical activity, and sleep, alongside biological mechanisms like hormonal regulation and body composition, may help clarify the pathways linking early weight gain to pubertal timing.

## Conclusion

Our study provides novel insights showing sex-specific associations between postnatal growth and puberty, using ultrasound as a marker of pubertal onset. Increased linear growth in early infancy was linked to earlier pubertal onset in both sexes, whereas weight gain from late infancy showed more consistent associations, particularly among girls. These associations were observed in children mostly within a normal weight range, indicating that early growth acceleration may affect pubertal timing in children with normal weight. This should be kept in mind when monitoring growth, even in healthy, term-born children.

## Data Availability

Restrictions apply to the availability of data generated or analyzed during this study to preserve patient confidentiality or because they were used under license. The corresponding author will on request detail the restrictions and any conditions under which access to some data may be provided.

## Abbreviations

AOR: adjusted odds ratio
APHV: age at peak height velocity
BGS2: Bergen Growth Study 2
BMI: body mass index
FSH: follicle-stimulating hormone
IOTF: International Obesity Task Force
IQR: interquartile range
LH: luteinizing hormone
Tanner PH: Tanner pubic hair
SD: standard deviation
SSF: subscapular skinfold
USB: ultrasound-measured breast stage
USTV: ultrasound-measured testicular volume

## References

[1] Parent AS, Teilmann G, Juul A, Skakkebaek NE, Toppari J, Bourguignon JP. The timing of normal puberty and the age limits of sexual precocity: variations around the world, secular trends, and changes after migration. Endocr Rev. 2003;24(5):668‐693. 14570750 10.1210/er.2002-0019

[2] Choi J-H, Yoo H-W. Control of puberty: genetics, endocrinology, and environment. Curr Opin Endocrinol Diabetes Obes. 2013;20(1):62‐68. 23183357 10.1097/MED.0b013e32835b7ec7

[3] Aksglaede L, Sørensen K, Petersen JH, Skakkebæk NE, Juul A. Recent decline in age at breast development: the Copenhagen puberty study. Pediatrics. 2009;123(5):e932‐e939. 19403485 10.1542/peds.2008-2491

[4] Biro FM, Greenspan LC, Galvez MP, et al Onset of breast development in a longitudinal cohort. Pediatrics. 2013;132(6):1019‐1027. 24190685 10.1542/peds.2012-3773 PMC3838525

[5] Eckert-Lind C, Busch AS, Petersen JH, et al Worldwide secular trends in age at pubertal onset assessed by breast development among girls: a systematic review and meta-analysis. JAMA Pediatr. 2020;174(4):e195881. 32040143 10.1001/jamapediatrics.2019.5881 PMC7042934

[6] Sørensen K, Aksglaede L, Petersen JH, Juul A. Recent changes in pubertal timing in healthy Danish boys: associations with body mass Index. J Clin Endocrinol Metab. 2010;95(1):263‐270. 19926714 10.1210/jc.2009-1478

[7] Biro FM, Khoury P, Morrison JA. Influence of obesity on timing of puberty. Int J Androl. 2006;29(1):272‐277; discussion 286–90. 16371114 10.1111/j.1365-2605.2005.00602.x

[8] Ong KK, Ahmed ML, Dunger DB. Lessons from large population studies on timing and tempo of puberty (secular trends and relation to body size): the European trend. Mol Cell Endocrinol. 2006;254-255:8‐12. 16757103 10.1016/j.mce.2006.04.018

[9] Graber JA . Pubertal timing and the development of psychopathology in adolescence and beyond. Horm Behav. 2013;64(2):262‐269. 23998670 10.1016/j.yhbeh.2013.04.003

[10] Deardorff J, Gonzales NA, Christopher FS, Roosa MW, Millsap RE. Early puberty and adolescent pregnancy: the influence of alcohol use. Pediatrics. 2005;116(6):1451‐1456. 16322170 10.1542/peds.2005-0542

[11] Prentice P, Viner RM. Pubertal timing and adult obesity and cardiometabolic risk in women and men: a systematic review and meta-analysis. Int J Obes (Lond). 2013;37(8):1036‐1043. 23164700 10.1038/ijo.2012.177

[12] Cheng TS, Day FR, Lakshman R, Ong KK. Association of puberty timing with type 2 diabetes: a systematic review and meta-analysis. PLoS Med. 2020;17(1):e1003017. 31905226 10.1371/journal.pmed.1003017 PMC6944335

[13] Benyi E, Sävendahl L. The physiology of childhood growth: hormonal regulation. Horm Res Paediatr. 2017;88(1):6‐14. 28437784 10.1159/000471876

[14] Dunger DB, Ahmed ML, Ong KK. Early and late weight gain and the timing of puberty. Mol Cell Endocrinol. 2006;254-255:140‐145. 16824679 10.1016/j.mce.2006.04.003

[15] Ahmed ML, Ong KK, Dunger DB. Childhood obesity and the timing of puberty. Trends Endocrinol Metab. 2009;20(5):237‐242. 19541497 10.1016/j.tem.2009.02.004

[16] Ong KK, Emmett P, Northstone K, et al Infancy weight gain predicts childhood body fat and age at menarche in girls. J Clin Endocrinol Metab. 2009;94(5):1527‐1532. 19240149 10.1210/jc.2008-2489

[17] Hvidt JJ, Brix N, Ernst A, Lauridsen LLB, Ramlau-Hansen CH. Size at birth, infant growth, and age at pubertal development in boys and girls. Clin Epidemiol. 2019;11:873‐883. 31572017 10.2147/CLEP.S217388 PMC6756829

[18] Wohlfahrt-Veje C, Tinggaard J, Juul A, Toppari J, Skakkebæk NE, Main KM. Pubarche and gonadarche onset and progression are differently associated with birth weight and infancy growth patterns. J Endocr Soc. 2021;5(8):bvab108. 34250379 10.1210/jendso/bvab108 PMC8262798

[19] Maisonet M, Christensen KY, Rubin C, et al Role of prenatal characteristics and early growth on pubertal attainment of British girls. Pediatrics. 2010;126(3):e591‐e600. 20696722 10.1542/peds.2009-2636 PMC5578444

[20] Aris IM, Perng W, Dabelea D, et al Analysis of early-life growth and age at pubertal onset in US children. JAMA Netw Open. 2022;5(2):e2146873. 35119461 10.1001/jamanetworkopen.2021.46873 PMC8817204

[21] Liimatta J, Jääskeläinen J, Mäntyselkä A, et al Accelerated early childhood growth is associated with the development of earlier adrenarche and puberty. J Endocr Soc. 2024;8(4):bvae026. 38425434 10.1210/jendso/bvae026 PMC10904224

[22] Wang Y, Dinse GE, Rogan WJ. Birth weight, early weight gain and pubertal maturation: a longitudinal study. Pediatr Obes. 2012;7(2):101‐109. 22434749 10.1111/j.2047-6310.2011.00022.x PMC3313082

[23] Bleil ME, Appelhans BM, Gregorich SE, et al Patterns of early life weight gain and female onset of puberty. J Endocr Soc. 2021;5(12):bvab165. 35274069 10.1210/jendso/bvab165 PMC8900195

[24] Marshall WA, Tanner JM. Variations in pattern of pubertal changes in girls. Arch Dis Child. 1969;44(235):291‐303. 5785179 10.1136/adc.44.235.291 PMC2020314

[25] Bordini B, Rosenfield RL. Normal pubertal development: part II: clinical aspects of puberty. Pediatr Rev. 2011;32(7):281‐292. 21724902 10.1542/pir.32-7-281

[26] Prader A . Testicular size: assessment and clinical importance. Triangle. 1966;7(6):240‐243. 5920758

[27] Juliusson PB, Bruserud IS, Oehme NHB, et al Deep phenotyping of pubertal development in Norwegian children: the Bergen Growth Study 2. Ann Hum Biol. 2023;50(1):226‐235. 37358552 10.1080/03014460.2023.2174272

[28] Bruserud IS, Roelants M, Oehme NHB, et al Ultrasound assessment of pubertal breast development in girls: intra- and interobserver agreement. Pediatr Radiol. 2018;48(11):1576‐1583. 29982956 10.1007/s00247-018-4188-7

[29] Diamond DA, Paltiel HJ, DiCanzio J, et al Comparative assessment of pediatric testicular volume: orchidometer versus ultrasound. J Urol. 2000;164(3 Part 2):1111‐1114. 10958754 10.1097/00005392-200009020-00048

[30] Bruserud IS, Roelants M, Oehme NHB, et al References for ultrasound staging of breast maturation, tanner breast staging, pubic hair, and menarche in Norwegian girls. J Clin Endocrinol Metab. 2020;105(5):1599‐1607. 32140730 10.1210/clinem/dgaa107 PMC7275631

[31] Oehme NHB, Roelants M, Saervold Bruserud I, et al Reference data for testicular volume measured with ultrasound and pubic hair in Norwegian boys are comparable with northern European populations. Acta Paediatr. 2020;109(8):1612‐1619. 31899821 10.1111/apa.15159

[32] Madsen A, Oehme NB, Roelants M, et al Testicular ultrasound to stratify hormone references in a cross-sectional Norwegian study of male puberty. J Clin Endocrinol Metab. 2019;105(6):1888‐1898. 10.1210/clinem/dgz094 31697832

[33] Madsen A, Bruserud IS, Bertelsen BE, et al Hormone references for ultrasound breast staging and endocrine profiling to detect female onset of puberty. J Clin Endocrinol Metab. 2020;105(12):e4886‐e4895. 32961560 10.1210/clinem/dgaa679 PMC7571452

[34] Balthasar MR, Roelants M, Brannsether-Ellingsen B, et al 2025. Supplemental data for: Early postnatal growth and pubertal timing: An ultrasound-based study of breast and testicular development. Figshare digital repository. 10.6084/m9.figshare.30316705. Deposited 17 October 2025 PMC12745745 41472892

[35] The Norwegian Directorate of Health . National guideline for Health Promotion and Preventive work in the child- and school health centres, and youth health service. 2017. Accessed 2025. https://www.helsedirektoratet.no/retningslinjer/helsestasjons-og-skolehelsetjenesten

[36] Juliusson PB, Roelants M, Nordal E, et al Growth references for 0-19 year-old Norwegian children for length/height, weight, body mass index and head circumference. Ann Hum Biol. 2013;40(3):220‐227. 23414181 10.3109/03014460.2012.759276

[37] Oehme NHB, Roelants M, Bruserud IS, et al Ultrasound-based measurements of testicular volume in 6- to 16-year-old boys—intra- and interobserver agreement and comparison with prader orchidometry. Pediatr Radiol. 2018;48(12):1771‐1778. 29980860 10.1007/s00247-018-4195-8

[38] Methlie P, Hustad SS, Kellmann R, et al Multisteroid LC-MS/MS assay for glucocorticoids and androgens, and its application in Addison's disease. Endocr Connect. 2013;2(3):125‐136. 23825158 10.1530/EC-13-0023 PMC3845685

[39] Oehme NHB, Roelants M, Bruserud IS, et al Low BMI, but not high BMI, influences the timing of puberty in boys. Andrology. 2021;9(3):837‐845. 33544961 10.1111/andr.12985

[40] Wankanit S, Mahachoklertwattana P, Pattanaprateep O, Poomthavorn P. Basal serum luteinising hormone cut-off, and its utility and cost-effectiveness for aiding the diagnosis of the onset of puberty in girls with early stages of breast development. Clin Endocrinol (Oxf). 2020;92(1):46‐54. 31705682 10.1111/cen.14124

[41] Marshall WA, Tanner JM. Variations in the pattern of pubertal changes in boys. Arch Dis Child. 1970;45(239):13‐23. 5440182 10.1136/adc.45.239.13 PMC2020414

[42] Cole TJ, Lobstein T. Extended international (IOTF) body mass index cut-offs for thinness, overweight and obesity. Pediatr Obes. 2012;7(4):284‐294. 22715120 10.1111/j.2047-6310.2012.00064.x

[43] Brannsether B, Roelants M, Bjerknes R, Juliusson PB. Waist circumference and waist-to-height ratio in Norwegian children 4-18 years of age: reference values and cut-off levels. Acta Paediatr. 2011;100(12):1576‐1582. 21627692 10.1111/j.1651-2227.2011.02370.x

[44] Brannsether B, Roelants M, Bjerknes R, Juliusson PB. References and cutoffs for triceps and subscapular skinfolds in Norwegian children 4-16 years of age. Eur J Clin Nutr. 2013;67(9):928‐933. 23632751 10.1038/ejcn.2013.91

[45] Zheng M, Lamb KE, Grimes C, et al Rapid weight gain during infancy and subsequent adiposity: a systematic review and meta-analysis of evidence. Obes Rev. 2018;19(3):321‐332. 29052309 10.1111/obr.12632 PMC6203317

[46] van Buuren S . Multiple imputation of discrete and continuous data by fully conditional specification. Stat Methods Med Res. 2007;16(3):219‐242. 17621469 10.1177/0962280206074463

[47] Papadimitriou A, Marakaki C, Papadimitriou DT. Growth variations with opposite clinical outcomes and the emerging role of IGF-1. Trends Endocrinol Metab. 2022;33(5):359‐370. 35331614 10.1016/j.tem.2022.02.004

[48] Flom JD, Cohn BA, Tehranifar P, et al Earlier age at menarche in girls with rapid early life growth: cohort and within sibling analyses. Ann Epidemiol. Mar. 2017;27(3):187‐193.e2. 10.1016/j.annepidem.2017.01.004 28215584

[49] Brix N, Ernst A, Lauridsen LLB, et al Childhood overweight and obesity and timing of puberty in boys and girls: cohort and sibling-matched analyses. Int J Epidemiol. 2020;49(3):834‐844. 32372073 10.1093/ije/dyaa056 PMC7394964

[50] Li Y, Ma T, Ma Y, et al Adiposity Status, trajectories, and earlier puberty onset: results from a longitudinal cohort study. J Clin Endocrinol Metab. 2022;107(9):2462‐2472. 35779008 10.1210/clinem/dgac395

[51] Aghaee S, Deardorff J, Quesenberry CP, Greenspan LC, Kushi LH, Kubo A. Associations between childhood obesity and pubertal timing stratified by sex and race/ethnicity. Am J Epidemiol. 2022;191(12):2026‐2036. 35998084 10.1093/aje/kwac148 PMC10144668

[52] Li W, Liu Q, Deng X, Chen Y, Liu S, Story M. Association between obesity and puberty timing: a systematic review and meta-analysis. Int J Environ Res Public Health. 2017;14(10):1266. 29064384 10.3390/ijerph14101266 PMC5664767

[53] Reinehr T, Roth CL. Is there a causal relationship between obesity and puberty? Lancet Child Adolesc Health. 2019;3(1):44‐54. 30446301 10.1016/S2352-4642(18)30306-7

[54] Loomba-Albrecht LA, Styne DM. Effect of puberty on body composition. Curr Opin Endocrinol Diabetes Obes. 2009;16(1):10‐15. 19115520 10.1097/med.0b013e328320d54c

